# The Role of Immunoproteasomes in Tumor-Immune Cell Interactions in Melanoma and Colon Cancer

**DOI:** 10.1007/s00005-022-00644-x

**Published:** 2022-01-22

**Authors:** Hanna Leister, Felix F. Krause, Rouzbeh Mahdavi, Ulrich Steinhoff, Alexander Visekruna

**Affiliations:** Institute for Medical Microbiology and Hygiene, Philipps-University Marburg, Marburg, Germany

**Keywords:** Immunoproteasome, Colitis-associated cancer, Melanoma, Tumor microenvironment, T cells

## Abstract

The participation of proteasomes in vital cellular and metabolic processes that are involved in tumor growth has made this protease complex an attractive target for cancer treatment. In contrast to ubiquitously available constitutive proteasome, the increased enzymatic activity of immunoproteasome is associated with tumor-infiltrating immune cells, such as antigen-presenting cells and T lymphocytes. In various tumors, an effective anti-tumor immunity is provided through generation of tumor-associated antigens by proteasomes, contributing crucially to cancer eradication by T lymphocytes. The knowledge regarding the role of immunoproteasomes in the communication between tumor cells and infiltrating immune cells is limited. Novel data suggest that the involvement of immunoproteasomes in tumorigenesis is more complex than previously thought. In the intestine, in which diverse signals from commensal bacteria and food can contribute to the onset of chronic inflammation and inflammation-driven cancer, immunoproteasomes exert tumorigenic properties by modulating the expression of pro-inflammatory factors. In contrast, in melanoma and non-small cell lung cancer, the immunoproteasome acts against cancer development by promoting an effective anti-tumor immunity. In this review, we highlight the potential of immunoproteasomes to either contribute to inflammatory signaling and tumor development, or to support anti-cancer immunity. Further, we discuss novel therapeutic options for cancer treatments that are associated with modulating the activity of immunoproteasomes in the tumor microenvironment.

## Introduction

The ubiquitin–proteasome system (UPS) has a central role in the selective degradation of intracellular proteins. More than 80% of eukaryotic proteins within a cell are degraded through the UPS to maintain cellular homeostasis and cell viability (Crawford et al. 2011). Consequently, the UPS regulates the levels and activity of numerous cellular proteins and, therefore, affects multiple cellular functions, such as cell cycle, apoptosis, inflammatory processes, DNA repair and transcription (Elliott et al. 2003). Dysfunction of the UPS is implicated in development of various diseases (Dahlmann 2007). An impairment of the UPS has been proposed as a common pathological feature among several autoimmune diseases and brain disorders, such as ischemia, epilepsy and neurodegenerative diseases, although the exact mechanisms remain poorly defined (Basler et al. 2014; Dantuma and Bott 2014; Schmidt et al. 2010). In cancer cells, the down-regulation of proteasome activity might lead to the escape of immune surveillance (Dahlmann 2007).

**Fig. 1:**
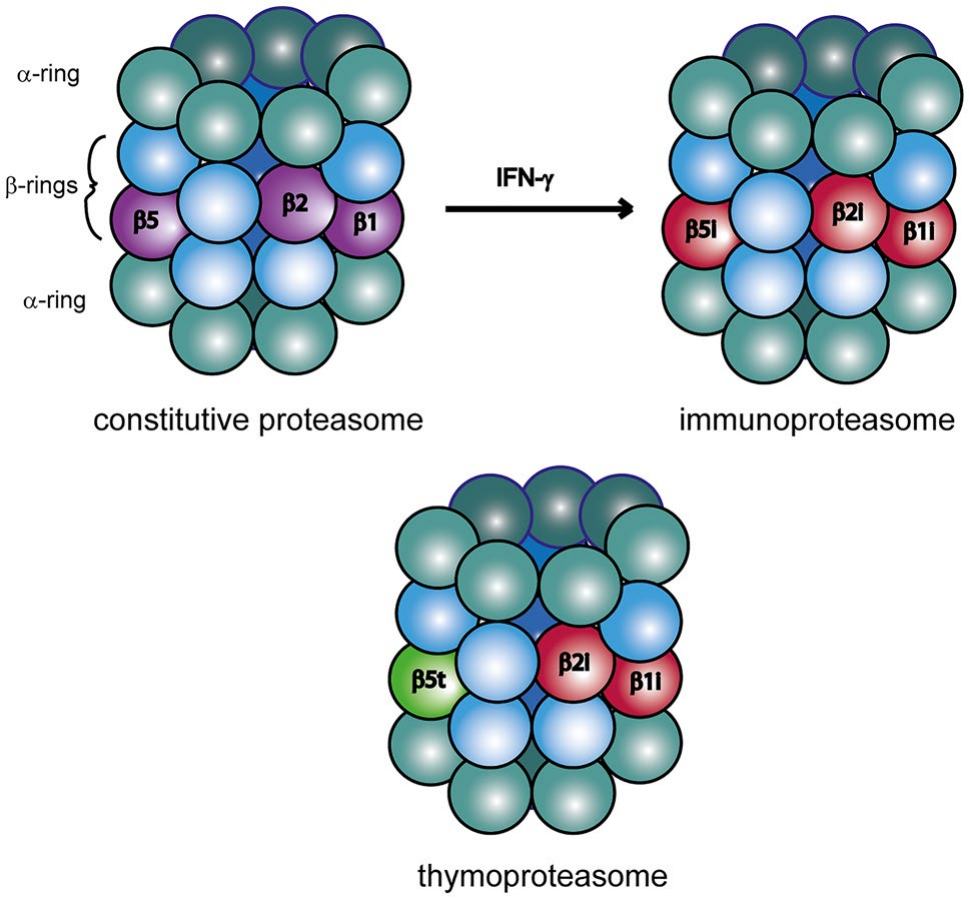
Three different types of proteasomes. The abundance of the majority of intracellular proteins is regulated through the ubiquitin proteasome system. The constitutive proteasome of eukaryotic cells is a multi-catalytic protease consisting of seven α and seven β subunits, of which three proteins, β1, β2, and β5 exhibit a catalytic activity. During infections with viruses or intracellular bacteria, mammalian constitutive proteasome is replaced by immunoproteasome (containing three de novo synthetized catalytic subunits β1i, β2i and β5i). This assembly process is induced via IFN-γ, leading to the optimized repertoire of antigenic peptides for MHC I class molecules. In addition, a specialized form of proteasome, thymoproteasome, containing the catalytic subunits β1i, β2i and β5t, is exclusively expressed in cortical thymic epithelial cells and is particularly efficient in producing low-affinity MHC class I peptides

Degradation of selected proteins by the proteasome is the central step of the ubiquitin–proteasome pathway. The proteasome is abundantly located in nuclei and cytoplasm of all eukaryotic cells to maintain cellular homeostasis (Reits et al. 1997). The 26S proteasome, a large multi-catalytic complex, is composed of a 20S core proteasome and two 19S regulatory units. The barrel-shaped 20S core proteasome consists of four stacked seven-membered protein rings. While the outer α-rings have scaffold-like function, the inner β-rings contain the enzymatic activity (Huber et al. 2012). The α-rings regulate the access to the core complex, whereby the α3 subunit is essential for sealing the central channel and stabilizing the closed state of proteasome (Groll and Huber 2003). The 19S protein complexes recognize ubiquitinated proteins and transfer them into the central catalytic cavity in an ATP-dependent manner. Following the selective degradation of protein substrates, short peptides are generated and subsequently presented on MHC class I molecules to CD8^+^ T cells (Kloetzel 2001). The three catalytic β-subunits (β1, β2 and β5), which are responsible for generating antigenic peptides, have distinct proteolytic activities: caspase-like (β1), trypsin-like (β2) and chymotrypsin-like (β5) activity (Fig. 1). Recently, a “bite and chew mechanism” was proposed, in which catalytic activities allosterically regulate each other (Kisselev et al. 1999). An initial cleavage of the peptide by chymotrypsin-like site (“bite”) stimulates the caspase-like site. Their activation accelerates further cleavage (“chewing”) of the fragments. The 20S core proteasome containing the catalytic subunits β1, β2 and β5 is called the constitutive or standard proteasome (Kruger et al. 2001). While constitutive proteasomes are ubiquitously expressed in all cell types of eukaryotes, there are other proteasome forms that are exclusively present in specific tissues (Kniepert and Groettrup 2014; Kuckelkorn et al. 2002). Apart from the constitutive proteasome, the best characterized type of proteasome is the immunoproteasome, which is optimized for efficient presentation of antigens on MHC I class molecules and is implicated in the differentiation of T cells via regulation of cytokine expression (Kalim et al. 2012; Kloetzel and Ossendorp 2004). Assembly of the immunoproteasome is induced through inflammatory cytokines such IFN-γ and TNF-α (Ebstein et al. 2013; Heink et al. 2005). Upon infection with viruses and intracellular bacteria, the three catalytic proteasome subunits are substituted in the infected tissue with the immunoproteasome subunits β1i (LMP2), β2i (MECL-1) and β5i (LMP7). Simultaneously, the 19S regulatory complex can be replaced with the 11S regulator composed of proteasome activators α (PA28α) and β (PA28β) (Basler et al. 2011; Kimura et al. 2015). Recently, cell-type specific proteasome subtypes, such as thymoproteasomes and spermatoproteasomes, were identified (Belote and Zhong 2009; Murata et al. 2007). The thymoproteasome is exclusively expressed in cortical thymic epithelial cells and is crucial for the selection of developing CD8^+^ T cells. This type proteasome contains two catalytic immunosubunits β1i and β2i, together with a specialized catalytic protein β5t, which is crucial for the functional activity of thymoproteasomes (Fig. 1). Mice deficient for β5t exhibit a strong reduction of CD8^+^ T cells with a markedly altered T-cell receptor repertoire (Nitta et al. 2010).

### Immunoproteasomes and Immune System

Immunoproteasomes are constitutively expressed in the cells of the immune system such as antigen-presenting cells (APCs) and T lymphocytes (McCarthy and Weinberg 2015). The altered catalytic activity of immunoproteasome subunits influences the quantity and quality of peptides presented by MHC class I molecules (Basler et al. 2013). Immunoproteasomes have a reduced caspase-like activity and enhanced chymotrypsin-like activity leading to more efficient generation of specific epitopes (Driscoll et al. 1993). According to current model, the immunoproteasome assembly is efficiently achieved by competitive integration of catalytic β subunits. The high affinity of immunosubunits to assembling proteasome complexes facilitates the replacement of constitutive proteasomes by immunoproteasomes during infection even in non-immune cells. A strong interaction of LMP7 and POMP (a chaperone that selectively binds to precursor subunits of the proteasome) promotes the immunoproteasome assembly and increases the proteasome amount in infected cells during infections (Heink et al. 2005), which is an essential step for clearance of intercellular pathogens by mammalian cells.

One of the main functions of the immunoproteasome is an efficient antigen processing for presentation on MHC class I molecules. Peptides generated by immunoproteasome show a higher binding affinity for the MHC class I complex (Aki et al. 1994). The lack of immunoproteasomes in mice reduces the CD8^+^ T-cell-mediated immune responses during influence virus, hepatitis B virus and lymphocytic choriomeningitis virus (LCMV) infections (Chen et al. 2001; Moebius et al. 2010; Robek et al. 2007). In addition, immunoproteasomes seem to be essential for survival of LCMV-specific CD8^+^ T cells in infected mice (Moebius et al. 2010). Furthermore, it was shown that mice with genetic deletion of immunoproteasomes are more susceptible to infections with intracellular bacteria and protozoan parasites. The combined deficiency of all three immunoproteasome subunits in mice abrogated the development of an effective host resistance against the human protozoan parasite *Trypanosoma cruzi*. Immunoproteasome-deficient mice exhibited significantly lower magnitude and quality of *T. cruzi*-specific CD8^+^ T-cell responses (Ersching et al. 2016). Moreover, during infection of mice with the intracellular bacterium *Brucella abortus*, a lower MHC I surface expression, an impairment of granzyme B and IFN-γ expression, as well as a reduced cytotoxic activity of CD8^+^ T lymphocytes was observed in the absence of immunoproteasomes (Guimaraes et al. 2018). In general, the immunoproteasome-mediated generation of peptides with higher binding affinity crucially impacts the availability and repertoire of epitopes for antigen presentation. Cells completely lacking immunoproteasomes have a restricted repertoire of presented peptides and a strong reduction of MHC class I surface expression compared to wild-type (WT) cells. Of note, the animals lacking immunoproteasomes reject skin transplants or splenic cells from WT mice, suggesting that cells in WT and immunoproteasome-deficient mice present a markedly different set of peptides (Kincaid et al. 2012; Toes et al. 2001). Recently, novel functions of immunoproteasomes in modulating a complex network of pro-inflammatory signaling pathways in APCs that are linked to autoimmunity, gut and neuro-inflammation, as well as to T helper (Th) cell differentiation and cytokine production have been proposed (Basler et al. 2013). Moreover, lack of immunoproteasomes is associated with decreased cellular ability to degrade oxidized proteins, showing so far unknown role for this protease complex in the cells (Ebstein et al. 2013; Seifert et al. 2010). Thus, in addition to immune functions, the immunoproteasome provides also protection against the accumulation of oxidatively damaged cellular proteins (Pickering et al. 2010).

### Immunoproteasome-Dependent Regulation of Inflammation-Driven Carcinogenesis in the Intestine

Immunoproteasome is crucially involved in mediating protective immunity against viral and bacterial antigens, but it is also implicated in the pathogenesis of several autoimmune diseases such as rheumatoid arthritis and multiple sclerosis (Basler et al. 2018; Muchamuel et al. 2009). We and others have shown that immunoproteasome activity in the inflamed intestine promotes the production of pro-inflammatory cytokines, such as IL-6, TNF-α, IL-17A and IL-23 (Basler et al. 2010; Schmidt et al. 2010; Visekruna et al. 2006). Because these cytokines are the integral part of the signaling network that synergistically activates NF-κB and STAT3 in colonic epithelial cells (De Simone et al. 2015), they are also crucially implicated in the onset of inflammation-associated carcinogenesis in the gut. IL-17A, IL-21, IL-22, TNF-α, IL-6 and IL-23 are also excessively produced in the early colonic lesions in patients with inflammatory bowel disease who have an increased risk for development of colon tumorigenesis (Karin 2009; Neurath 2014; West et al. 2015). Thus, the cytokine network abundantly secreted in the inflamed areas of gastrointestinal tract contributes to the induction of oncogenic transcription factors in colonocytes, which promotes cell survival and uncontrolled proliferation. Interestingly, the simultaneous neutralization of IL-17A and TNF-α abrogating NF-κB signaling pathways, or IL-22 and IL-6 inhibiting STAT3-mediated signaling impairs the mitogenic effects on colorectal cancer cells (De Simone et al. 2015). In mice, the deletion of IL-17A or IL-23 was sufficient to significantly reduce the number and size of tumors in experimental model of colitis-associated cancer (Grivennikov et al. 2012; Hyun et al. 2012). Importantly, the specific inhibition of the immunoproteasome subunit LMP7, but also the non-specific blockade of proteasomes by bortezomib, suppresses the expression of these inflammatory mediators and prevents the development of acute colitis in mice (Kalim et al. 2012; Schmidt et al. 2010). The immunoproteasome-specific inhibitor, ONX 0914 was effective in suppressing the onset of inflammation-driven cancer even when applied in a therapeutic setting when the tumor size was macroscopically visible in mice (Koerner et al. 2017; Vachharajani et al. 2017). In recent study, we addressed the role of immunoproteasomes in the azoxymethane (AOM)-dextran sodium sulfate (DSS) model of colitis-associated cancer (CAC) in mice with combined deficiency of all three immunoproteasome subunits, LMP2, LMP7 and MECL-1. Remarkably, immunoproteasome-deficient triple-knockout mice were not susceptible to the development of CAC, since no visible tumors were detected in these mice (Leister et al. 2021). In addition, in the lamina propria, a negligible expression of pro-inflammatory chemokines CXCL1, CXCL2 and CXCL3, as well as cytokines that contribute to the CAC progression such IL-6, TNF-α, IL-17A and IL-23 was observed. Of note, we also found an upregulation of immunoproteasome-regulated pro-tumorigenic chemokines in patients with ulcerative colitis, who have a high risk for development of colorectal cancer (Leister et al. 2021). These findings suggest that in humans a similar mechanisms may lead to the recruitment of neutrophils and other innate immune cells that promote the damage in the gut epithelial cells and contribute to the onset of tumorigenesis. Collectively, these data have identified the immunoproteasome as a key regulator of pro-tumorigenic signaling networks in the inflamed gut, leading to the onset of colorectal carcinoma. Interestingly, one study demonstrated that LMP7 inhibition was effective not only in a model of inflammation-driven cancer, but also in APC^Min/+^ mice, a preclinical model that closely resembles familial adenomatous polyposis in humans, suggesting that immunoproteasomes might be also involved in promoting inflammation-independent carcinogenesis (Koerner et al. 2017). The components of the UPS such as immunoproteasomes might be directly involved in the regulation of proliferation and differentiation, as well as in pro-apoptotic signaling pathways in colorectal cancer cells (Voutsadakis 2008). An effective targeting strategy for colorectal cancer in future may be a specific blockade of immunoproteasomes with small compounds, which, in contrast to non-specific proteasome inhibitors, could reduce therapeutic side effects, as the cells expressing constitutive proteasomes are not targeted.

In conclusion, accumulating evidence strongly suggests that immunoproteasomes regulate the activity of infiltrated immune cells in the inflamed gut and promote the development of CAC. Thus, novel immunoproteasome-specific inhibitors should be tested in future clinical studies to optimize the treatment of rectal and colon adenocarcinomas.

### The Role of Immunoproteasomes in the Tumor Microenvironment of Melanoma and Other Solid Tumors

**Fig. 2:**
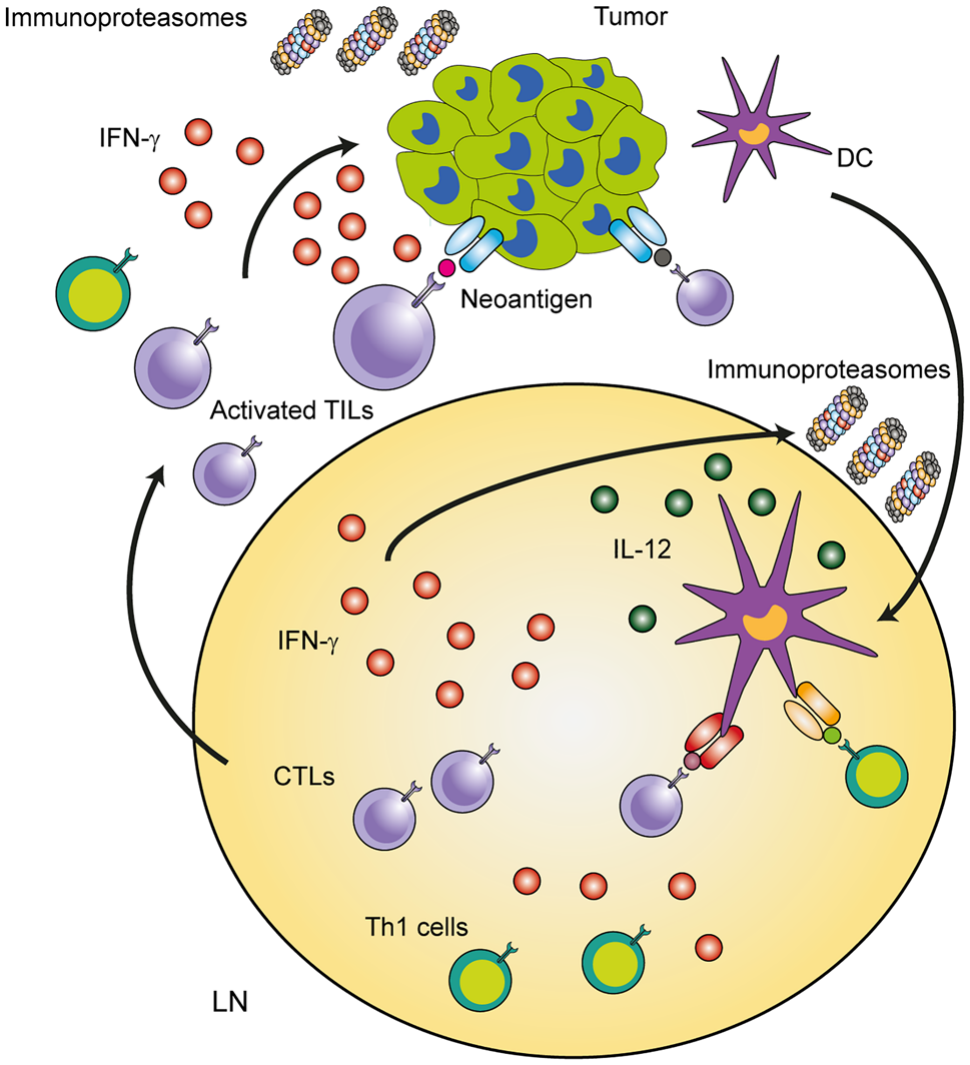
Immunoproteasomes support the anti-tumor immunity in melanoma. MHC class I-bound antigens derived from tumor cells are presented by professional antigen-presenting cells such as dendritic cells (DCs) in the tumor-draining lymph nodes (LNs). The repertoire of peptides presented to CD8^+^ T cells is dependent on the activity of immunoproteasomes in tumor cells, which can be enhanced by IFN-γ produced by activated tumor-infiltrated lymphocytes (TILs). Some neo-antigens presented by tumor cells are recognized by T cells, which will induce strong cancer-specific T-cell responses. In addition to their role in generating peptides for presentation on MHC I class molecules, immunoproteasomes regulate the expression of IL-12 and other cytokines in DCs. Activated DCs produce high levels of IL-12 in LNs, thus promoting differentiation of cytotoxic T lymphocytes (CTLs) and Th1 cells. CTL- and Th1-derived IFN-γ enhances, in turn, the expression of immunoproteasomes in DCs, which optimizes the IL-12 secretion. Immunoproteasomes exhibit several crucial functions that potentiate the anti-tumor immunity against melanoma

Infiltration of cytotoxic T lymphocytes (CTLs) and Th1 cells into the microenvironment of solid tumor is a pivotal step in recognizing tumor-associated antigens and in preventing the tumor growth and progression (Ritter and Greten 2019). Further, immunoproteasomes are involved in shaping the repertoire of neo-antigens and the activation of antigen-specific CD8^+^ T-cell responses in the tumor microenvironment. Recent studies have revealed that the local production of IFN-γ and high expression of the immunoproteasome subunits LMP2, LMP7 and MECL-1 strongly correlate with the abundance of tumor-infiltrating lymphocytes, and with better survival of melanoma patients (Hugo et al. 2016; Kalaora et al. 2020; Leister et al. 2021). Of note, the overexpression of immunoproteasomes in human melanoma cell lines led to the generation of more immunogenic repertoire of tumor peptides and better lysis of tumor cells by co-cultured autologous tumor-infiltrating lymphocytes (Kalaora et al. 2020). These findings support the hypothesis that the high expression of immunoproteasomes in solid tumors might result in superior killing of tumor cells by CTLs due to alterations in antigen repertoire, and possibly by increased generation and recognition of neo-antigens. Importantly, the enhanced activity of immunoproteasomes correlated also with a better response of melanoma patients to immune-checkpoint inhibitor therapy such as anti-CTLA4 and anti-PD1 (Kalaora et al. 2020). Furthermore, in a murine model of melanoma, in which the melanoma cell line B16-F10 was able to express immunoproteasomes, while the tumor recipient mice were immunoproteasome-deficient, an increased tumor volume with impaired anti-tumor immunity was observed (Leister et al. 2021). These data demonstrate that immune cells, such as dendritic cells, Th1 cells and CTLs, which express high amount of immunoproteasomes in the tumor microenvironment, are essential for an effective immunity against melanoma, and probably also against other solid tumors such as lung cancer (Spits and Neefjes 2016). Immunoproteasomes are known to regulate the production of IL-12 by APCs, which ultimately leads to enhanced infiltration of lymphocytes in the tumor tissue and a better differentiation of tumor-specific Th1 cells and CTLs in tumor-draining lymph nodes (Fig. 2). Notably, by inoculating melanoma cells into WT animals and mice lacking immunoproteasomes, we observed that immunoproteasomes were strongly induced in tumor cells derived from WT mice, but not in that from immunoproteasome-deficient animals (Leister et al. 2021). These results demonstrate that Th1 lymphocytes and CTLs, surrounding the tumors and producing large amounts of IFN-γ, force cancer cells to enhance their immunoproteasome expression and activity. This significantly affects the repertoire of tumor antigens and might lead to more efficient presentation of neo-antigens derived from driver mutations (Fig. 2). Consequently, this would lead to a better elicitation of an antigen-specific immune response, and even to improved response to checkpoint inhibitor therapy. Thus, simultaneous activation of the immunoproteasome activity in solid tumors and in immune cells of the tumor microenvironment might result in improved cancer immunotherapies against various solid cancers of different origin. In accordance, one study in patients with non-small cell lung cancer (NSCLC) revealed that the immunoproteasome expression in tumor cells influences the responsiveness to immunotherapy (Tripathi et al. 2016). Likely, due to enhanced activity of immunoproteasomes in tumor cells, a more diverse tumor antigen pool is efficiently recognized by CTLs, resulting in a better outcome of disease. Accordingly, the deficiency of immunoproteasome expression was associated with a poor outcome in patients with NSCLC. Thus, the high immunoproteasome amount in solid tumors, such as melanoma and NSCLC, is linked to an increased diversity of tumor antigens, as well as to better responsiveness to immune-checkpoint therapy, and most importantly, also to better overall survival of patients.

Collectively, tumors have developed many strategies to avoid immune surveillance and to minimize the production of neo-antigens. Hence, a better understanding and a deeper knowledge of multiple interaction between tumor and immune cells in the tumor microenvironment is needed to develop therapeutic strategies for facilitating generation of more immunogenic tumor antigens. This might be achieved by enhancing the activity of immunoproteasomes in both, tumor and immune cells.

## Concluding Remarks

The flexibility and fine-tuning of the UPS is important for immune cells to adopt to inflammation, viral infections and cancers. It is well known that pro-inflammatory factors such as IFN-γ synergistically induce all three subunits of immunoproteasome to optimize the presentation of antigens on MHC class I molecules. In addition to shaping the T-cell repertoire for CTLs to better recognize tumor antigens, immunoproteasomes also modulate the cytokine production in APCs, T-cell differentiation, and even the secretion of chemokines and other factors by cancer cells. Novel results have revealed that the immunoproteasome is a crucial player in promoting colon tumorigenesis, and thus a potential molecular target for treatment of colorectal cancer. In contrast, the anti-tumorigenic properties of immunoproteasomes have been described for melanoma and other cancer types. Various aspects of proteasomal activities appear to be implicated in the multiple crosstalk of cancers with immune cells. Therefore, a better understanding of the immunoproteasome activity in different cancer types and in the immune cells of tumor microenvironment will be essential for developing targeted therapeutic approaches aiming at improving anti-tumor immunity. The utilization of pharmacological inhibition and modulation of immunoproteasome function in various cancer types, but also in the context of adoptive cell therapy, may shed light on novel therapeutic strategies to halt the progression of malignant neoplastic diseases.
